# Facing the possibility of consciousness in human brain organoids

**DOI:** 10.1016/j.patter.2025.101365

**Published:** 2025-09-12

**Authors:** Christopher Wood, Hao Wang, Wei-Jun Yang, Yongmei Xi

**Affiliations:** 1 MOE Laboratory of Biosystem Homeostasis and Protection, College of Life Sciences, Zhejiang University, Hangzhou 310058, China; 2 The University of Oxford, Oxford, UK; 3 Department of Neurosurgery of Second Affiliated Hospital and School of Brain Science and Brain Medicine, Key Laboratory for Biomedical Engineering of Education Ministry, Zhejiang University School of Medicine, Hangzhou, Zhejiang 310058, China; 4 NHC and CAMS Key Laboratory of Medical Neurobiology, MOE Frontier Science Centre for Brain Research and Brain Machine Integration, Key Laboratory of Precise Treatment and Clinical Translational Research of Neurological Diseases, School of Brain Science and Brain Medicine, Zhejiang University, Hangzhou, Zhejiang 310058, China; 5 Department of Neurosurgery, Centre for Genetic Medicine, the Fourth Affiliated Hospital of School of Medicine, and International School of Medicine, International Institutes of Medicine, Zhejiang University, Yiwu 322000, China

**Keywords:** human brain organoids, consciousness, neural correlates, neuroethics, precautionary principle, ethics

## Abstract

Human brain organoids (HBOs) have emerged as transformative models for neurodevelopment and disease, yet ethical concerns persist regarding their potential to develop consciousness. Since 2020, a growing cohort of neuroscientists and philosophers has dismissed these concerns as unscientific, citing limited structural complexity, absence of bodily integration and environmental interaction, and a prevailing neuroscientific consensus against the feasibility of any, or any near-future, emergence of HBO consciousness, thus challenging any suggested revisions of ethical guidelines and safeguards. We argue that this dismissal is premature. Drawing on neuroscientific benchmarks, comparisons to the developing human brain, contemporary theories of consciousness, and principles of natural developmental progression, we question the basis for selectively excluding consciousness from among HBOs’ expanding functional repertoire. We caution against enshrining such skepticism into dogma or using it to defer ethical engagement. Instead, we advocate for proactive, ongoing assessment of the moral implications of advancing HBO capabilities.

## The bigger picture

From embryo research to cloning to the 2015 gene-editing scandal, science has a history of high-profile developments that ignite public debate and divide opinion. Human brain organoids (HBOs), models of developing brain tissue grown in culture from human stem cells, may soon enter the same ballpark. For some, it is impossible not to imagine science-fiction-like images of brains grown in the lab, especially with examples of organoids already playing the computer game Pong and producing electroencephalography (EEG) patterns comparable to those of a human fetus. These powerful tools for studying human brain development and disease, with their ability to mimic key features, including the development of sophisticated neural networks, raise a particularly provocative question: could they ever develop consciousness?

In answering this question, many scientists have planted their flags firmly in the “no” camp, citing the immature stage of HBO development, lack of vasculature, and absence of certain brain structures and cell types, rightly framing their current state as far from the size, structure, and complexity of the human brain. Barriers to greater size and complexity in HBOs, however, are rapidly eroding, and skepticism toward HBO consciousness may be predicted to erode along with them. In seeking to avoid hype, and perhaps also to counter calls for extra ethical oversight or legislative control that could slow the rapid expansion of HBO research, we consider whether scientists may have gone too far in framing consciousness as a non-issue. This perspective calls for a more balanced view, one that neither sensationalizes nor dismisses the possibility. As HBO technology advances, the stakes extend well beyond the lab, shaping ethical guidelines, legislation, and public trust.

## Teaser

The authors challenge recent claims that the emergence of consciousness in human brain organoids (HBOs) remains implausible in the near term. Drawing on neuroscientific evidence, developmental parallels, and theories of consciousness, they argue that confident skepticism is premature. As barriers to HBO complexity diminish, selectively excluding consciousness from other likely functional advances lacks strong justification. Ethical engagement must remain active and, while avoiding overstatement, should also avoid premature dismissal of the possibility of HBO consciousness.

## Introduction

Every aspect of thought and emotion is rooted in brain structure and function, …—Steven Pinker^1^

When I read philosophy or neuroscience papers about consciousness, I don’t get the sense we’re any closer to understanding it than we were 50 years ago.—Stuart J. Russell^2^

Organoids are 3D cell cultures generated in the laboratory to replicate key structural and functional aspects of their target organs.^3^ Take the heart, for example. When induced pluripotent stem cells (iPSCs) are differentiated into cardiac lineages, they spontaneously self-organize into 3D structures that recapitulate the native tissue architecture, cellular composition, and spatial organization of the *in vivo* heart. As structure begets function, upon maturation, this structural organization enables the emergence of the heart’s core functional behavior: spontaneous rhythmic contractions.^4^ Based on earlier development of self-organized cortical tissues derived from embryonic stem cells,^5^ in 2013, researchers reported their prior creation of iPSC-derived human brain organoids (HBOs).^6^ Like their cardiac predecessors, these brain organoids demonstrated remarkable self-organizing capacity. Guided to differentiate into neural lineages, these former iPSCs began spontaneously coordinating themselves into 3D assemblies, mirroring many aspects of the structure and functional networks of the developing *in vivo* brain,^6^^,^^7^ thus rendering them suitable models for the study of early neurodevelopmental processes and neurological disease.

Upon their development, much philosophical and neuroscientific discussion ensued about where this may lead. Just as cardiac organoids, once sufficiently mature, advance from form to fundamental function (spontaneous beating), might sufficiently mature HBOs spontaneously develop aspects of one of the brain’s most fundamental functions: consciousness? If this occurred, how would this affect the moral status of HBOs and the ethical boundaries of their use in research?^8^^,^^9^^,^^10^^,^^11^ Perhaps fearing that consciousness-related concerns and related restrictions might stifle scientific progress, neuroscientists and philosophers have mobilized, both individually and collectively, in many post-2020 publications to defend HBO research. In many of these publications, they claim a neuroscientific consensus that dismisses anything but a far distant potential for HBO consciousness as biologically implausible, a solution chasing a problem assuredly not to emerge for decades, if ever.^11^^,^^12^^,^^13^^,^^14^^,^^15^^,^^16^^,^^17^^,^^18^ In doing so, they have positioned any current reassessment of HBO use criteria, or calls for additional safeguards, as unnecessary.

This perspective challenges such skeptical assertions. With reference to neurobiological evidence and philosophical principles, we will frame confident skepticism of HBO consciousness as increasingly biologically untenable, given the accelerating pace of such neuroscientific progress in organoid development, and epistemically overconfident, given the enduring opacity surrounding consciousness, its fundamental nature, and the precise conditions required for its emergence. Overall, we question whether there is any genuine empirical or theoretical justification that exists for the assumption that consciousness, among other observed brain functions, would necessarily remain absent as HBOs advance toward greater complexity and maturity. In this, we maintain a deliberately narrow focus. While most authors acknowledging the possibility of HBO consciousness would then go on to subsequently address (1) what specific form(s) such consciousness may take and (2) the extent to which those forms could be said to hold moral relevance, we do not attempt to do so here. While our argument implies that a maturing HBO consciousness would likely progress beyond rudimentary forms into morally significant territory, our primary aim is more foundational: to challenge the basis of the many recent expressions of consciousness skepticism before they solidify into dogma. In this way, we seek to ensure that these critical questions remain vibrant within mainstream neuroscience rather than being dismissed as mere exaggerated philosophical speculation.

## Assessing the likelihood of consciousness in HBOs

Consciousness, including its precise nature and underlying neural mechanisms, remains incompletely understood. Direct and definitive methods for its measure are similarly elusive. The proliferation of competing theories in consciousness research continues to fuel debates in neuroscience and philosophy.^19^^,^^20^ Nevertheless, many definitively worded papers have recently emerged promoting strong skepticism toward HBO consciousness. We begin by analyzing their position.

### The skeptical stance: Structural and functional limitations

In a published debate over HBO consciousness, Stanford geneticist Greely represents the skeptical position well, strongly critiquing the characterization of HBOs as “mini-brains.” While acknowledging that HBOs exhibit limited differentiation, with some features resembling certain *in vivo* brain regions, he emphasizes their stark anatomical differences, noting the absence of key structures such as a bilaterally symmetric cortex with distinct lobes, a cerebellum, or a hippocampus, thereby rightly underscoring that HBOs lack the architectural complexity of a mature cerebral cortex.^16^

Many skeptics continue to outline such stark contrasts. They emphasize the comparably primitive state of current HBOs, typically containing tens of millions of neurons, far fewer than the 86 billion in the adult human brain,^8^ reaching, at their largest size, the dimensions of a pinkie fingernail. In this, while the most advanced current HBO neuron count and complexity surpasses that of insects such as cockroaches, it remains significantly below that observed in adult mice.^16^ Further growth is noted as hindered by critical limitations, particularly the lack of vascularization and essential supporting cells, such as microglia, in most current models.^8^

Building on these observations, interdisciplinary collaborations between neuroscientists and philosophers have resulted in many recent papers published with strongly worded conclusions. These typically assert one of three positions: (1) current scientific evidence does not support the possibility of HBOs *ever* achieving the structural or functional complexity necessary for genuine consciousness or self-awareness^12^ (de Jongh’s systematic review of organoid ethical issues,^12^ for example, references nine papers from biomedical researchers and ethicists under this claim); (2) dismissing concerns over HBO consciousness as “biologically unfounded”^14^ and asserting that consciousness will not emerge *for the foreseeable future*^15^; or (3) even if consciousness were to arise, it would necessarily be primitive, rudimentary, and thus of little moral significance, where any suggestion of a “human-like” consciousness is rejected, with proponents asserting there is “no reason to think it may ever exist.”^16^ This skeptical view is not universal. Some scientists and ethicists have expressed a notably less skeptical stance regarding HBO consciousness,^21^^,^^22^^,^^23^^,^^24^ and others offer a balanced appraisal.^12^ Nonetheless, direct assertions of a general skeptical consensus concerning the consciousness potential of HBOs persist, at least in Western contexts. A striking example is the 2023 survey of HBO scientists conducted by Lavazza and Chinaia, which concluded that “the possibility of consciousness emerging in a model of the human brain” is “not yet considered pertinent by the scientific community.”^25^

The skeptical position often further emphasizes the disembodied isolation of HBOs from their external environment, a limitation framed as equally fundamental to the development of consciousness. The absence of sensory input, effector output, and, consequently, any capacity for environmental interaction has thereby been presented as a “fatal flaw” in arguments for HBO consciousness.^15^ Sophisticated brain networks, such as those associated with consciousness, are argued to typically require both sensory input and environmental engagement.^17^ The mereological fallacy may also be invoked in this context, whereby it is considered fallacious to attribute properties of the whole organism, such as consciousness, to individual brain parts in isolation.^26^ Thus, the disembodied state of HBOs serves as another key premise for concluding that concerns about potential HBO consciousness remain biologically and theoretically unsupported.^11^^,^^14^^,^^15^^,^^17^

Given these considerations, many publications thereby dismiss concerns about HBO consciousness as products of media sensationalism, science-fiction-inspired public misconceptions, or exaggerated optimism or attribute them to a philosopher’s speculative thought experiments, ultimately rejecting such claims as lacking scientific credibility.^12^^,^^14^^,^^15^^,^^16^^,^^17^^,^^18^ Consequently, advocates of HBO consciousness are charged with sparking unnecessarily and baseless ethical anxiety.^16^^,^^17^^,^^18^ Neuroscientists working directly with HBOs have therefore pushed back against such unrealistic media-driven narratives and philosophical speculation, as they see them, asserting a strong consensus that consciousness is implausible in any near-term organoid model.^17^^,^^25^ This view has been formally endorsed by authoritative bodies, including the German National Academy of Sciences, which concluded that HBO research raises no immediate ethical or legal concerns, given their assessment that consciousness will not emerge in the foreseeable future.^27^^,^^28^ Similarly, the International Society for Stem Cell Research (ISSCR) exempts HBOs from certain ethical oversight protocols, stating there is “no biological evidence to suggest any issues of concern, such as consciousness or pain perception” that would necessitate stricter review.^28^^,^^29^^,^^30^

### Proponent counterarguments: Neural complexity and theoretical accommodation

Against skeptical claims, proponents highlight a striking degree of morphological and functional maturity in HBOs. In these, they highlight clear parallels between HBOs and the developing *in vivo* human brain. HBOs exhibit complex neural networks, synchronized oscillatory activity (e.g., gamma, alpha, and delta waves), and functional connectivity that closely mirror preterm infant electroencephalography (EEG) patterns.^3^^,^^8^^,^^9^^,^^31^^,^^32^^,^^33^^,^^34^ These observations have led some researchers to assert that HBOs “mimic the development of the *in vivo* human brain with high degrees of accuracy,”^31^ recapitulating cellular-level neurogenesis, and where local patterning produces brain regions of proper relative positioning and spatial orginization relative to their *in vivo* counterparts, , at least at a local level if not yet at a global or macro scale.^7^^,^^32^

In 2019, Velasco et al. emphasized the significance of such HBO breakthroughs. In their work, they explicitly noted that previous assumptions regarding developmental constraints, which expressed doubt that the processes of human brain development could ever occur outside the context of embryogenesis, were now revealed as demonstrably false. With the advent of HBOs came the definitive demonstration that the development of the central nervous system no longer requires an embryonic context.^35^ We speculate that current skepticism citing constraints precluding consciousness in HBOs may follow a similar trajectory, with future advances likely to overturn present objections.

The field certainly seems poised for just such advances that would lead to greater HBO maturity, size, and complexity. Microfluidic platforms are extending HBO longevity and enabling longer-term cultivation.^7^^,^^33^ Ongoing advances in fibrous microscaffolds,^32^^,^^36^ including 3D electroconductive scaffolds and mesh electrodes, are further stimulating HBO development.^7^^,^^32^ Approaches to generate HBO capillary networks are emerging,^28^ potentially allowing functional vascularization.^28^^,^^36^^,^^37^ Success in the development of microglia-containing organoids^38^ and modes of distinct cortical layer formation^32^^,^^39^ are combining to increase key parallels to early postnatal transitions.^40^ These advances are expected to soon enable significant increases in the size, structural complexity, and functional sophistication of HBOs.^3^^,^^17^^,^^21^ As this occurs, arguments against HBO consciousness based upon insufficient complexity will surely grow progressively weaker, yielding ever-diminishing returns.

Secondly, the idea that the development of sophisticated brain networks, such as those associated with consciousness, typically requires sensory input and environmental interaction for their development^11^ is directly challenged by the evidence that potentially related networks are already beginning to develop in HBOs.^41^ This includes brain areas strongly implicated in consciousness, such as those of the prefrontal cortex and occipital lobe.^42^ Markers for such regions are already present in today’s HBOs, though currently relatively scattered and lacking the macro structure and layered architecture of their *in vivo* counterparts. Despite this, the preclusion of the emergence of functional consciousness as these networks mature and potentially gain resolution upon future development seems largely unsupported.

The question of whether current neuroscientific theories of consciousness accommodate the idea of HBO consciousness is also highly pertinent. With one notable exception (the temporal theory of consciousness, see below), these may be argued to not only accommodate but also to predict the likely development of HBO consciousness. In this, the lack of environmental or bodily interactions is not viewed as a fundamental barrier. For instance, the integrated information theory (IIT) allows for the possibility of an internal world of conscious experience independent of external input, drawing parallels from phantom limb pain and the dreaming brain as examples of self-contained “islands of awareness” or consciousness streams unshaped by sensory perception.^8^^,^^21^^,^^43^ Some proponents of the IIT have suggested that the primary neural correlate of consciousness, localized in a posterior cortical “hot zone,” contains structural features that support the high integration necessary for consciousness as measured by a maximal Φ (where Φ measures how much information a system generates as a unified whole; the more integrated and irreducible this information is, the higher the potential for consciousness, according to the IIT).^24^ The IIT is then suggested to posit an exceptionally low threshold for minimal consciousness, one that does not require a large number of neurons. This opens the door to the possibility that even strikingly simple systems, including HBOs, could exhibit some level of consciousness, despite their limited complexity and maturity when compared to the *in vivo* human brain.^24^ As noted above, the temporal theory of consciousness, which is based upon how the brain tracks the state of its environment, presents an exception but remains inaccessible for most HBO contexts due to its requirement of the incorporation of inputs from both the body and surroundings.^20^

Similarly, the global workspace theory (GWT) proposes that consciousness emerges when processed information is globally “broadcast” via excitatory neuronal activity across widely distributed neural networks.^20^ Thus, the presence of a large-scale signal broadcast involving the stimulation of widely distributed networks linked to the formation of neuronal circuits, already present to some extent and likely to soon further develop in current HBOs, may render consciousness skepticism ever more unsubstantiated. This is particularly compelling given that these patterns emerge spontaneously in HBOs, suggesting an intrinsic capacity for conscious-like organization (see later argument on natural progression). Overall, if empirical observations increasingly validate the presence of dynamically coordinated oscillatory and synchronous activity among and between neural populations,^20^ we suggest that it will become increasingly difficult to justify the claim that consciousness development remains highly improbable, especially when these neural dynamics mirror those theorized to underlie conscious awareness.

However, we must emphasize here that our argument applies specifically to multi-region or “unguided”^44^ HBOs, sometimes called “whole-brain organoids,”^8^ not to single region or “guided” HBOs. The latter, guided by additional patterning factors, exclusively models only specific regions of the brain, such as the forebrain, midbrain, brainstem, cerebral cortex, or pituitary.^8^^,^^44^ Critically, unless experimentally networked together to form “fusion organoids” or “assembloids,” thus reestablishing inter-regional connectivity,^45^^,^^46^^,^^47^^,^^48^ these single-region HBOs may lack the integrated architecture that some current neuroscientific frameworks identify as likely essential for consciousness. Therefore, here, our primary focus remains on multi-region/unguided/whole-brain organoids or assembloids that may align with the IIT’s requirement for integrated information (the IIT’s Φ > 0 requirement) and/or functional network broadcasting (the GWT prerequisite). Consequently, we suggest ethical discussions about organoid consciousness should prioritize multi-region models, where such regional integrations come the closest to mirroring the biological substrates of consciousness as we currently understand them.

### Natural progression as an argument for HBO consciousness

Despite the above, one could argue that a yet stronger case for HBO consciousness development rests on simpler grounds. The current debate finds proponents and skeptics talking past each other: skeptics selectively emphasizing differences to deny its possibility, while advocates selectively emphasize similarities to declare its likelihood, both demonstrating how easily the question may be begged. Fundamentally, this is not about selective biological analogies but about the question of by what clear principle we can justify the overall assumption that consciousness, among many other neural capabilities, would fail to emerge, given further development and maturity of HBO systems.

In this, it is important to recognize that consciousness, if it emerges, is not primarily a product of scientific intervention. Scientific efforts are employed in actively improving scaffolds, optimizing nutrient substrates, and removing barriers to HBO growth, in doing so supporting, rather than causing, any potential emergence of consciousness. The potential itself fundamentally depends on the intrinsic capacity contained within the HBOs themselves.

The foundation of just such a capacity therefore seems already present and active in current HBOs. Given the opportunity and optimal conditions, HBOs get busy with deliberate pre-programmed purposes, rapidly resulting in the development of the ordered, complex neuronal structures and synaptic networks that mirror processes observed *in vivo*. At this stage, neuroscientists are largely relegated to the role of observers, witnessing the unfolding complexity, one that is clearly directional and purposeful—the very concept that justifies HBO-based research as able to reveal previously hidden aspects of early human neurodevelopment.

In this HBO context, once development is externally initiated (e.g., from iPSCs), control shifts from extrinsic to intrinsic regulatory processes. Subsequent development becomes predominantly self-organized and intrinsically directed. At this stage, the HBOs develop in an internally directed manner, continuing independently toward maturity unless actively hindered. It therefore seems clear that an intrinsic active potency for consciousness already likely exists within developing HBOs as part of their inherent ontological nature. Consciousness should thus be considered inherently possible due to the intrinsic programming and inherent capacity and nature of HBOs, as they model human brain development and realize the potential they already carry.

This compels us to re-examine the foundational assumptions underlying HBO consciousness skepticism. Here, we invert the traditional burden of proof. Rather than requiring advocates to demonstrate that consciousness *will* emerge, we challenge skeptics to justify their assertion that it *will not*. While neither assertion remains invulnerable to philosophical counter, the simple question remains that, viewing what appears to be at least the beginnings of consciousness-related networks, on what basis can we confidently assert that these networks will not be continued to their completion to functionality? What definitive empirical or theoretical justification exists for claiming that consciousness, among many other brain functions, would invariably remain absent as these developmental processes advance toward greater complexity and maturity? Associated mechanisms and theories can surely be presented by the skeptic or advocate alike, but no principled scientific basis seems firmly established that justifies this selective exclusion of consciousness from the growing repertoire of neural capabilities that HBOs demonstrably exhibit.

We must remember that the entire rationale for organoid research hinges on their ability to faithfully replicate the structure and function of native organs.^12^ HBOs betray their growing validity in this regard by modeling the very organ uncontroversially recognized as the primary biological substrate of consciousness. Though existing in a profoundly artificial environment, unguided HBOs may reasonably be expected to develop consciousness as a natural outcome of their development. Despite their unnatural context, these remain fundamentally human brain cells, executing the intrinsic genetic program that naturally gives rise to consciousness *in vivo*.

This raises a pressing question of *timing*. As already discussed, while some neuroscientists, perhaps reluctantly, acknowledge the theoretical possibility of HBO consciousness, they consistently relegate this prospect to the far distant future. However, recent studies show that HBOs can already attain transcriptional profiles comparable to a human fetal brain up to 24 weeks post-conception, though not yet beyond.^8^ This brings them remarkably close to a critical developmental threshold of the 24–28 week period when thalamocortical connections form in fetuses—the very structures hypothesized to enable conscious pain perception, for example.^49^

Therefore, while current limitations in size and complexity must be acknowledged, three fundamental considerations challenge any confident assertion that consciousness will not emerge in the foreseeable, perhaps even imminent, future.

(1) Consciousness remains fundamentally unresolved: the field broadly acknowledges that we lack both a definitive framework for understanding consciousness and clear criteria for its emergence.
(2) Seeds of neural correlates are already present: HBOs seem to be already actively in the process of developing the very neuronal architectures and dynamic networks implicated in conscious processing.
(3) Technological barriers are temporary: advances, perhaps exponential, in organoid research are likely to systematically overcome previous limits to growth and functional complexity.

Given this triad, neuroscientists who categorically and confidently exclude HBO consciousness^12^^,^^14^^,^^15^^,^^16^^,^^17^^,^^18^ seem to be engaging in precisely the type of speculative overreach they criticize in consciousness advocates. The very premise that legitimizes HBO research, the faithful recapitulation of the development processes, functions, and trajectories of the *in vivo* human brain, contains a notable function that typically emerges midway through this trajectory: consciousness. Though consciousness represents just one facet among many, its possible emergence carries profound implications that could ethically upset the whole apple cart. This positions skeptical neuroscientists^12^^,^^14^^,^^15^^,^^16^^,^^17^^,^^18^ in a paradoxical bind: they are in danger of celebrating HBOs as valid models of brain development (justifying continued research) yet dismissing the possibility of consciousness, a feature inherent to the very trajectory they claim HBOs replicate. In doing so, they could be said to embrace the model’s power while resisting its consequences. Whether ethical and regulatory concerns have unduly influenced this stance (consciously or unconsciously!) remains a critical question.

## Conclusion

Given that HBOs model *in vivo* brain development as part of their essential purpose, the biological emergence of some degree of consciousness occurring at a reasonably early stage of HBO development, as it does *in vivo*, would not be profoundly surprising. We therefore argue that the burden of proof lies with the skeptic, not the advocate. Strong dismissive claims regarding HBO consciousness, such as assertions claiming that there is “no possibility” of its imminent occurrence,^15^ rendering its concept “biologically unfounded” over the near future,^14^ or suggesting that there is no evidence that it will *ever* become possible,^12^ appear, at the very least, speculative and overconfident. Even within such skeptical literature, there is widespread acknowledgment of the profound opaqueness in our current neuroscientific understanding of consciousness, the very foundation upon which many of these denials rest. Many of these same sources similarly concede that existing limitations to growth, maturity, and complexity are likely temporary barriers, soon to be overcome as the field advances. Thus, the categorical dismissal of HBO consciousness appears not only premature but internally inconsistent with the field’s own evidence and admissions of uncertainty and progress.

Critically, skeptics often also recognize that HBOs already exhibit functional neural networks, some of which may plausibly correlate with conscious processes.^15^ If HBOs continue to mature and develop, displaying high integration necessary for consciousness (per IIT) or larger-scale signal broadcasts (per GWT), the neural dynamics currently theorized to underlie consciousness skepticism may become yet more difficult to justify. Conversely, to confidently proclaim selective examples of the current neurological differences between HBOs and *in vivo* brains as *necessarily* rather than simply *potentially* consciousness precluding in the future seems equally inconsistent. At a minimum, such assertions should be tempered, considering the many epistemic limitations and the precautionary principle.^50^

Against the above, concerns about proponent overreach remain valid. Even if HBO networks achieve maturity levels comparable to those implicated in *in vivo* consciousness, this alone would not confirm phenomenological experience, nor any degree of moral equivalency or relevance. Such systems might represent “empty houses”—functionally analogous but devoid of experiential content. As Kosik suggests, they could constitute a pre-configured state “present in the absence of any experience,” merely prepared to encode experience when it arises.^15^

Nevertheless, we argue that the emergence of consciousness in HBOs remains a biologically plausible scenario, one that cannot be confidently ruled out by appeals to current structural limitations, disembodiment, or neuroscientific consensus. While this paper remains largely agnostic regarding the specific potential character of such consciousness (i.e., to what extent it might be considered human-like) and its wider ethical implications, our simpler aim here is to maintain that strong denials of the possibility of any near- to mid-term HBO consciousness emergence should not be accepted by the scientific community or general public uncritically, nor used to circumvent or postpone urgent ethical deliberation as HBO research advances.

## Acknowledgments

We offer grateful thanks to Professor Katrien Devolder of the Philosophy Department of the University of Oxford and Professor Bai Ge of the Medical School of Zhejiang University for valuable mentorship and support during this writing process. Also, we offer genuine thanks to the four anonymous reviewers whose comments have helped strengthen this paper considerably.

## Author contributions

C.W. was responsible for conceptualization and writing the original draft under the supervision of W.H. W.-J.Y. and Y.X. then joined C.W. and W.H. for the final review and editing.

## Declaration of interests

The authors declare no competing interests.

## Declaration of generative AI and AI-assisted technologies in the writing process

After completing the writing of this work, the authors used DeepSeek and occasionally ChatGPT for some sections in order to conduct a final language check and polish. No scientific or philosophical content was generated with AI. Language changes were kept to a minimum. After using this tool/service, the authors reviewed and edited the content as needed and take full responsibility for the content of the publication.
